# Chaining Differential Reinforcement of Compliance and Functional Communication Training to Treat Challenging Behavior Maintained by Negative Reinforcement

**DOI:** 10.3390/bs15070891

**Published:** 2025-06-30

**Authors:** Emily L. Ferris, Alexandra R. Howard, Eleni Baker, Daniel J. Hodge, Andrew R. Craig, Henry S. Roane, William E. Sullivan

**Affiliations:** Golisano Center for Special Needs, SUNY Upstate Medical University, Syracuse, NY 13210, USA; ferrise@upstate.edu (E.L.F.); howardal@upstate.edu (A.R.H.); bakerele@upstate.edu (E.B.); hodgedan@upstate.edu (D.J.H.); craiga@upstate.edu (A.R.C.); roaneh@upstate.edu (H.S.R.)

**Keywords:** functional communication training, differential reinforcement of compliance, treatment chaining, chained schedule, preference, challenging behavior, demand fading

## Abstract

Differential reinforcement of compliance (DRC) and functional communication training (FCT) are two effective treatments for escape-maintained behavior. They each, however, have unique limitations. This study aimed to replicate and extend past work by isolating the effects of each treatment and assessing for treatment preference. FCT produced larger reductions in challenging behavior and lower levels of compliance relative to DRC, which produced elevated levels of both compliance and challenging behavior. Additionally, all participants preferred FCT to DRC. Overall, challenging behavior was low and compliance was high when both treatments were embedded within a chained schedule, and these reductions were maintained throughout fading.

## 1. Introduction

Challenging behaviors, such as aggression and self-injury, are prevalent among individuals with autism spectrum disorder (ASD) and can lead to significant disruption in daily life (Cohen et al., 2011). These behaviors profoundly impact the individuals who engage in them and their caregivers, increasing the risk of harm and stress while negatively affecting academic progress, social relationships, treatment outcomes, and caregiver confidence in managing their child’s behavior (Stevenson et al., 2019; Durand, 2021). Among the various functions of challenging behavior, escape-maintained behavior—engaging in challenging behavior to escape from aversive situations—represents a significant subset. Melanson and Fahmie (2023) reported that 25.90% of cases in the published literature were maintained by escape (i.e., social-negative reinforcement), highlighting the need for targeted interventions to address these behaviors. Effective and sustainable treatments for escape-maintained behavior must increase prosocial behavior, reduce challenging behavior, and be generalizable to the natural environment.

Two evidence-based interventions for escape-maintained challenging behavior are differential reinforcement of compliance (DRC) and functional communication training (FCT), both of which are classified as differential reinforcement of alternative behavior (DRA) procedures (Geiger et al., 2010). Although DRC and FCT can be implemented using either differential negative reinforcement of alternative behavior (DNRA) or differential positive reinforcement of alternative behavior (DPRA)—depending on the maintaining function of the target behavior and the programmed consequence—in the current context, both are used as DNRA procedures, where the alternative responses (e.g., compliance or functional communication) are reinforced by escape from demands. DRC and FCT replace challenging behavior with alternative prosocial responses, such as compliance (DRC) or functional communication (FCT), to access escape from non-preferred stimuli (Geiger et al., 2010). Piazza et al. (1996) demonstrated that combining DRC, which reinforces task completion, with demand fading effectively reduced escape-maintained challenging behavior to near-zero levels while increasing compliance, supporting its use in addressing challenging behavior. Similarly, Carr and Durand (1985) introduced FCT to teach individuals to use functionally equivalent communication responses (FCRs) to request escape from demands. Both DRC and FCT are established and highly effective interventions for reducing escape-maintained challenging behavior (Hanley & Tiger, 2011; Tiger et al., 2008).

Although DRC and FCT are widely used and effective, each has limitations in addressing escape-maintained behaviors. DRC increases compliance, but does not necessarily promote functional communication, whereas FCT encourages appropriate communication, but does not inherently increase compliance. Moreover, both interventions often require dense reinforcement schedules during the initial stages to produce treatment effectiveness, which can be impractical for caregivers and difficult to implement in naturalistic settings (Greer et al., 2016; Nuhu & Pence, 2021). These limitations emphasize the need for strategies that increase the sustainability and generalizability of these interventions.

Lalli et al. (1995) addressed the individual limitations of DRC and FCT by evaluating response chaining in the context of FCT for escape-maintained challenging behavior. In their study, three participants were initially taught to engage in an alternative, prosocial communication response (i.e., an FCR) to escape demands while challenging behavior was placed on extinction. Although this intervention reduced challenging behavior, it did not increase compliance, as participants continued to avoid demands by engaging in the FCR. To address this limitation, Lalli et al. (1995) implemented a chained schedule of reinforcement in which escape was contingent on a trained verbal response (i.e., FCR) following the completion of an increasing number of responses. Their findings demonstrated that response chaining with FCT and extinction effectively increased compliance while maintaining low levels of challenging behavior. However, their study did not directly assess the independent effects of DRC, leaving it unclear whether DRC alone could have promoted functional communication or whether its combination with FCT provides unique benefits. Additionally, Lalli et al. (1995) did not account for participant intervention preference across DRC and FCT, a factor that has since been recognized as critical in the selection and long-term success of behavioral interventions (Hanley, 2010).

Current best practice guidelines emphasize the importance of incorporating individual preferences when selecting behavioral interventions, noting that socially valid treatments are more likely to be maintained over time (Hanley, 2010). Social validity, or the acceptability and perceived effectiveness of a treatment from the recipient’s perspective, is crucial to long-term treatment adherence (Pelton et al., 2024). Hanley et al. (2005) demonstrated that individuals may exhibit clear preferences for different intervention components, highlighting the need to assess participant preference alongside treatment efficacy. Since DRC and FCT target different alternative behaviors commonly associated with escape-maintained challenging behavior—compliance and functional communication, respectively—understanding individual preferences for these interventions may not only contribute to their success but may also promote the sustainability of and adherence to treatment procedures. By integrating treatment preference assessments into intervention selection, practitioners can tailor behavioral interventions to the needs of the individual, ultimately increasing feasibility and promoting sustained behavior change.

By combining DRC and FCT within a chained schedule, the limitations of each intervention may be addressed, potentially encouraging compliance and functional communication while reducing the reliance on dense reinforcement schedules. Building on the work of Lalli et al. (1995), this study seeks to replicate and extend their findings by systematically evaluating DRC and FCT in isolation before integrating them within a chained schedule. Specifically, this study aimed to (a) assess participant preferences between DRC and FCT, (b) evaluate the individual effects of DRC and FCT on escape-maintained challenging behavior, and (c) examine the impact of their combination in a chained schedule with extinction.

## 2. Method

### 2.1. Participants and Settings

Three children referred to an outpatient clinic for the assessment and treatment of severe challenging behavior participated. Allan, a 10-year-old boy, was diagnosed with reactive attachment disorder and an intellectual disability. Emily, a 13-year-old girl, and Liam, a 9-year-old boy, both had an autism spectrum disorder (ASD) diagnosis. All participants were primarily vocal communicators and demonstrated the ability to follow multi-step instructions. The participants’ target behaviors included aggression, self-injurious behavior (SIB), oppositional statements, and disruptions. Emily had previous exposure to FCT for a tangible function, and Liam had previous exposure to FCT for synthesized escape-tangible functions. Allan had no previous exposure to FCT interventions. To our knowledge, no participants had history with DRC within the clinical setting.

Sessions were conducted in a 3 m by 3 m padded therapy room with a one-way observation window. Session materials included toys, an iPad, and discrimination cards to signal treatment conditions. Additional items specific to each participant’s needs were also provided, as identified in the pre-experimental assessments (see below). Across participants, an average of 5.50 sessions occurred per day (range, 1.00–11.00 sessions).

### 2.2. Response Measurement and Data Collection

Target behaviors included *oppositional statements*, defined as any statement refusing instruction, delaying compliance, complaining about demands, using vulgar or inappropriate language (i.e., threats to others, swearing, or suicidal, sexual, or race-related statements), or using an argumentative tone; *disruptions*, defined as throwing objects, kicking or hitting the walls or furniture, turning over furniture, or throwing demand materials; *aggression*, defined as hitting, punching, kicking, pushing, or pinching others; and *SIB*, defined as head banging and hitting oneself. Allan’s target behaviors included oppositional statements, disruptions, aggression, and SIB. Emily’s target behavior was oppositional statements. Liam’s target behaviors included oppositional statements and disruptions.

Observers collected data behind a one-way mirror using a computer-based data collection system (BDataPro; Bullock et al., 2017). Interobserver agreement of combined target behaviors (IOA) was assessed using an exact-interval method and was collected for a minimum of 30.00% (range, 30.33–76.25%) of sessions across all phases of the study. A second observer collected data for an average of 54.58% of sessions across all participants. Overall, IOA was 95.95% across participants. The mean IOA for Allan’s combined target behaviors was 98.26% (range, 90.00–100.00%), with a mean of 99.84% for aggression (range, 93.33–100.00%), a mean of 99.85% for disruptions (range, 96.67–100.00%), a mean of 93.35% for oppositional statements (range, 60.00–100.00%), and a mean of 100.00% for SIB. The mean IOA for Emily’s target behavior was 95.67% (range, 73.33–100.00%). The mean IOA for Liam’s combined target behaviors was 91.84% (range, 63.34–100.00%), with a mean of 90.94% for disruptions (range, 50.00–100.00%) and a mean of 92.75% for oppositional statements (range, 72.41–100.00%).

Compliance was defined as the participant engaging in the response associated with the demand (e.g., picking up toys when the demand was “clean up your toys”) following either the verbal prompt or model (i.e., the first two steps of three-step prompting). Responses that were physically guided were not marked as compliance. IOA of compliance was assessed using an exact-interval method and was collected for a minimum of 30.00% (range, 30.33–76.25%) of sessions across all phases of the study. A second observer collected data for an average of 54.58% of sessions across all participants. Overall, IOA was 95.38% across participants. The mean IOA for Allan was 96.24% (range, 70.00–100.00%), 94.72% for Emily (range, 73.33–100.00%), and 95.18% for Liam (range, 80.00–100.00%).

For all participants, the FCR was defined as touching a card associated with the reinforcer (i.e., a “break/toys” card). The FCR was not available under baseline or DRC alone conditions. IOA of FCRs was assessed using an exact-interval method and was collected for a minimum of 30.00% (range, 30.33–76.25%) of sessions across all phases of the study. A second observer collected data for an average of 54.58% of sessions across all participants. Overall, IOA was 94.56% across participants. The mean IOA for Allan was 90.64% (range, 53.33–100.00%), 97.35% for Emily (range, 86.21–100.00%), and 95.70% for Liam (range, 80.65–100.00%).

### 2.3. Pre-Experimental Assessments

#### 2.3.1. Tangible Preference Assessment

Following the procedure outlined by Fisher et al. (1992), a paired-choice preference assessment was conducted to identify highly preferred items for each participant. Preferred items included an iPad, Transformer toys, and pegs for Allan; an iPad and stuffed animals for Emily; and an iPad and Play-Doh for Liam. These preferred items were incorporated into subsequent phases of the study, including the functional analyses and treatment conditions.

#### 2.3.2. Functional Analysis

Functional analyses were conducted with all participants to identify maintaining variables for their target behaviors. Conditions used in the functional analyses were selected through a caregiver interview. For Allan, a functional analysis, modeled after Iwata et al. (1982/1994), was initially implemented, including attention, tangible, escape, and toy–play control conditions. Demands in the escape condition included spelling, tracing, and math worksheets. Following elevated levels of responding in both the tangible and escape conditions, a pairwise functional analysis (Iwata et al., 1994) was conducted with a combined tangible-escape condition and a toy–play control condition. For Emily, a functional analysis, modeled after Iwata et al. (1982/1994), was implemented, including attention, tangible, escape, alone, and toy–play control conditions. Demands in the escape condition were multiplication. For Liam, a functional analysis modeled after Iwata et al. (1994) was conducted with an attention condition, an escape condition, a toy–play control condition, and a combined tangible-escape condition. Demands used in the escape and tangible-escape conditions were cleaning up tangible items. During these sessions, target behaviors were reinforced by providing access to consequences corresponding to the condition (e.g., access to attention, escape, and/or tangibles) for 20 s. Sessions lasted 5 min and were repeated to identify and confirm the functions of the participant’s target behaviors.

## 3. Procedures

### 3.1. Treatment Preference Assessment

Participants completed a concurrent treatment preference assessment prior to the treatment comparison phase. This was conducted prior to the treatment comparison because the preferred treatment identified by the assesment was used as a reinforcer during the chained schedule. The treatment preference assessment included a single session, beginning with exposure trials for each treatment condition, followed by 10 choice trials. Treatment conditions were signaled by colored cards. Exposure trials were conducted to ensure that participants experienced equal exposure to each of the contingencies and their respective colored cards. During DRC exposure trials, the session therapist presented a discrimination card (blue for Allan, pink for Liam, and yellow for Emily, hereafter referred to as the DRC-card), and compliance with one demand (e.g., tracing and math worksheets for Allan, verbal multiplication for Emily, and cleaning up tasks for Liam) produced access to a 20 s break and preferred toys. During FCT exposure trials, the session therapist presented a discrimination card (yellow for Allan and Liam, and blue for Emily, herein referred to as FCT-card), and emitting an FCR resulted in access to a 20 s break and preferred toys. FCRs for all participants were a card touch (i.e., a “break/toys” card). During choice trials, participants faced a table where both DRC- and FCT-cards were placed equidistantly and were allowed to select one, determining the treatment implemented for that trial. The position of the discrimination cards (i.e., which color was on the left or the right) was counterbalanced across trials. The overt selection response of selecting a card (i.e., a concurrent chain arrangement; Hanley et al., 1997) was used to ensure that participants were responding based on preference, as opposed to the response effort of the initial response. Emily experienced 10 exposure trials for each treatment condition prior to choice trials. Allan experienced 40 exposure trials for each treatment condition, due to having no previous history with either condition. Due to a procedural fidelity error, Liam initially experienced choice trials prior to exposure sessions. Upon identifying the error, Liam experienced 10 exposure trials for each treatment condition, followed by 10 new choice trials.

### 3.2. Treatment Comparison (DRC vs. FCT)

Baselines in this condition were identical to the participant’s functional analysis test conditions (i.e., a tangible-escape condition). Specifically, for Allan and Liam’s baseline sessions, they had unrestricted access to preferred toys with demand materials present. Session therapists delivered instructions using three-step prompting. Contingent on target behavior, the therapist said “okay, you can have a break,” while simultaneously removing the demand materials and allowing 20 s of access to toys. For Emily’s baseline sessions, she had unrestricted access to preferred toys. To begin a trial, the session therapist would issue a vocal math demand (e.g., “what is two times three?”). Contingent on target behavior, the therapist said “okay, you can keep playing,” and stepped away from Emily, while allowing 20 s of access to toys. Baseline sessions lasted five minutes and were repeated three to five times per participant. Baseline data collection required a minimum of three data points, with progression to the treatment comparison phase contingent on a stable or countertherapeutic trend.

During treatment comparison, 5 min DRC and FCT sessions were randomly alternated in a multielement design, with an equal number of sessions conducted for each condition. Target challenging behaviors were placed on extinction across both treatments. In DRC sessions, the blue or pink card was present, and the session therapist removed access to the toys and delivered demands using a three-step prompting procedure (vocal instruction, model prompting, and physical prompting). Compliance with one demand was differentially reinforced with a break from demands and access to preferred toys for 20 s (i.e., an FR 1 schedule). Response effort for demands was approximately equated for difficulty within participants. In FCT sessions, the yellow card was present, and access to toys was removed and demands were delivered. The emission of an FCR was differentially reinforced with a break from demands and access to preferred toys for 20 s (i.e., an FR 1 schedule). The treatment comparison phase utilized a multielement design and continued until clear differentiation between DRC and FCT was observed. Differentiation was defined as at least 50.00% of the data points for one treatment exceeding the upper confidence limit of the other treatment. If this criterion was met, participants proceeded to the Chained Schedule (described below). If differentiation between treatments was not seen, a reversal design was implemented to demonstrate experimental control. This occurred for Allan only.

### 3.3. Chained Schedule

The effects of the chained schedule were evaluated using a reversal design. Baseline was identical to the baseline condition described above. Following baseline, DRC and FCT were evaluated in a two-component chained schedule with the first component associated with the DRC contingency and signaled by the DRC-card, and the second component associated with FCT and a corresponding discrimination card. The session therapist presented the DRC-card, removed access to preferred toys and delivered demands using the three-step prompting procedure. Compliance with one demand resulted in removing the DRC-card and presenting the FCT-card. Emitting an FCR was then reinforced with access to a break from demands and preferred tangibles for 20 s. Before progressing to demand fading, baseline conditions were re-established, and stability criteria were applied.

### 3.4. Demand Fading

In the final phase, the response ratio required under the DRC component was systematically increased before access to the FCT component was provided. Initially, compliance with one demand was required to access the second component of the chained schedule (i.e., FCT). This ratio increased one at a time following two consecutive sessions with a greater than 80% reduction in target challenging behavior. Likewise, if the participant engaged in target challenging behavior that produced a less than an 80% reduction across two consecutive sessions, the requirement was decreased by one. This process continued until compliance with five demands was required in the DRC component as a demonstration of this process.

## 4. Results

**Figure 1:**
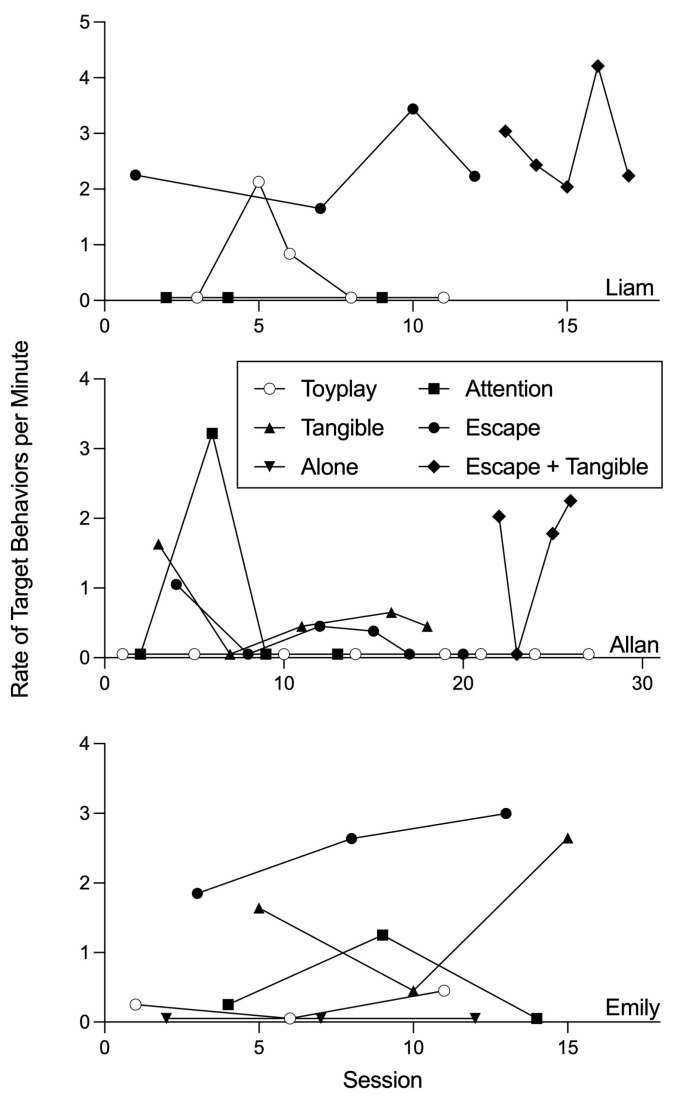
Functional analyses of target behaviors for all participants.

**Figure 2:**
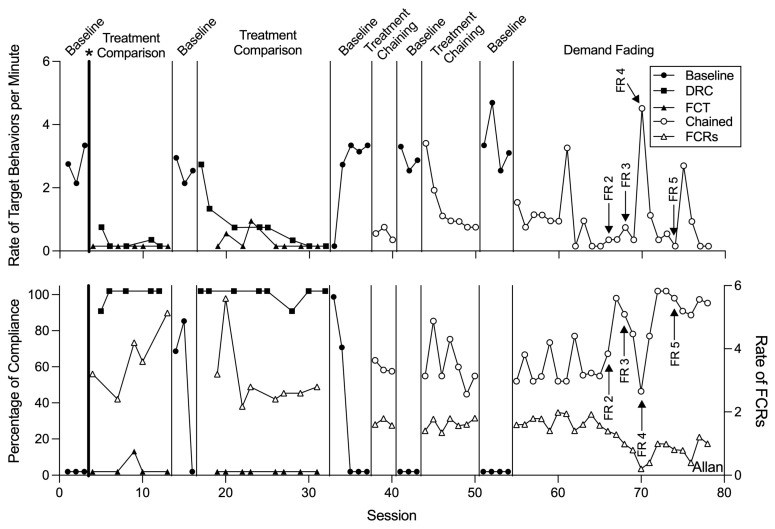
Rate of target behavior per minute and percentage of demands complied with for Allan. Note. Asterisk and bolded phase change line denote when the treatment preference assessment was conducted; FR = fixed ratio; DRC = differential reinforcement of compliance; FCT = functional communication training; FCR = functional communication response.

**Figure 3:**
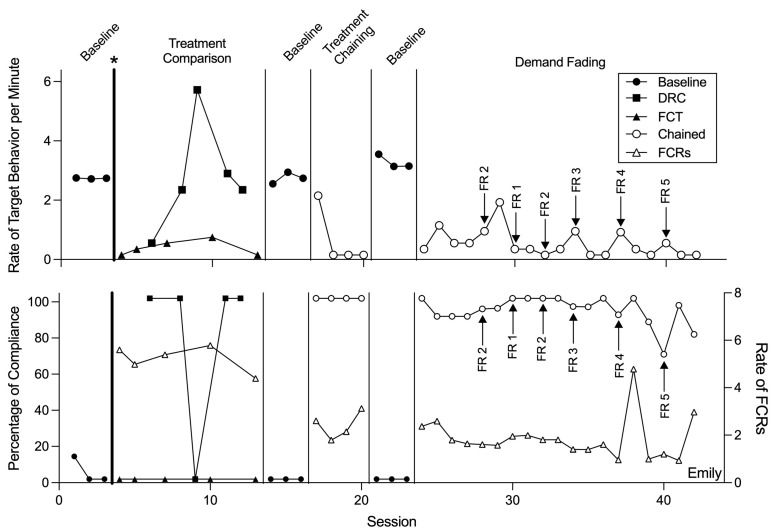
Rate of target behavior per minute and percentage of demands complied with for Emily. Note. Asterisk and bolded phase change line denote when the treatment preference assessment was conducted; FR = fixed ratio; DRC = differential reinforcement of compliance; FCT = functional communication training; FCR = functional communication response.

**Figure 4:**
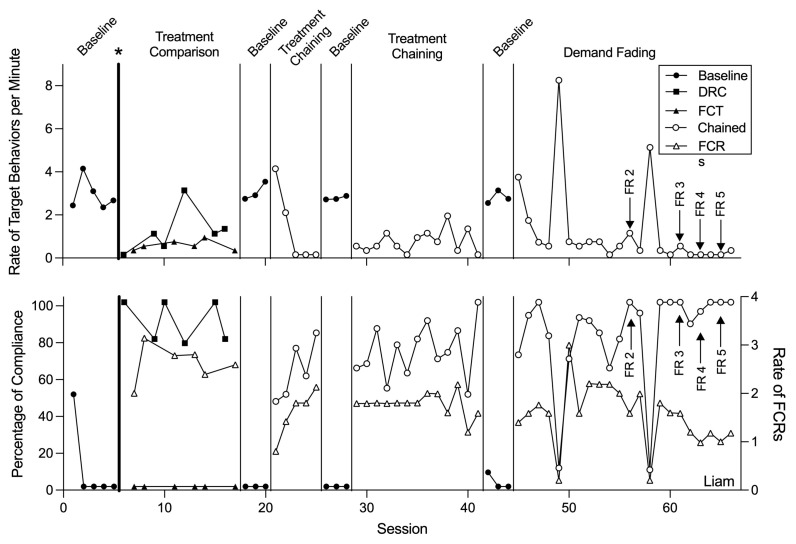
Rate of target behavior per minute and percentage of demands complied with for Liam. Note. Asterisk and bolded phase change line denote when the treatment preference assessment was conducted; FR = fixed ratio; DRC = differential reinforcement of compliance; FCT = functional communication training; FCR = functional communication response.

Functional analyses (Figure 1) indicated that all participants’ target challenging behaviors were multiply maintained by escape from demands and access to preferred tangible items. Figure 2, Figure 3 and Figure 4 display the results of the treatment comparison, chained schedule, and demand fading for Allan, Emily, and Liam, respectively. During initial baseline sessions, all participants engaged in target challenging behavior with little to no compliance (except for session one of baseline with Emily and Liam). Allan engaged in an average of 2.59 challenging behaviors per minute (range: 1.99–3.19 responses per minute [rpm]) and did not comply with any demands. Emily engaged in an average of 2.59 challenging behaviors per minute (range: 2.57–2.60 rpm) and complied with an average of 4.17% of demands throughout baseline. Liam engaged in an average of 2.79 challenging behaviors per minute (range: 2.20–4.00 rpm) and complied with 10.00% of demands throughout baseline.

Prior to moving on to the treatment comparison, the treatment preference assessment was conducted with each participant. As noted in Section 3.1, Liam initially experienced choice trials prior to exposure sessions. For these initial choice trials, Liam selected the FCT-card nine times, and the DRC-card one time. Upon identifying the procedural error, Liam experienced 10 exposure trials for each treatment condition, followed by 10 new choice trials. During choice trials that followed exposure trials, all participants selected the FCT-card for all 10 trials.

During the treatment comparison, FCT resulted in decreases in Allan’s target challenging behavior to an average of 0.09 rpm, but compliance with an average of only 0.85% of demands. DRC led to increased compliance, with an average of 98.29% of demands resulting in compliance; however, challenging behavior during DRC occurred at variable rates, averaging 0.50 rpm. Reversals to baseline led to a return to initial levels of both challenging behavior and compliance.

For Emily, FCT resulted in a decrease in target challenging behavior to an average of 0.24 responses per minute. DRC led to 100% compliance with demands, except for session nine, where compliance dropped to 0%. Challenging behavior under DRC occurred at a higher rate than with FCT, averaging 2.62 responses per minute. Reversals to baseline resulted in a return to initial levels of both target behaviors and compliance.

Liam’s FCT treatment phase resulted in an average of 0.43 responses per minute for challenging behavior. Under DRC, he demonstrated 89.62% compliance with demands, with challenging behavior occurring at an average of 1.09 responses per minute. Similar to the other participants, reversals to baseline led to a return to initial levels of both challenging behavior and compliance.

In the chained schedule analysis, Allan exhibited a substantial reduction in target challenging behaviors and an increase in compliance. Allan’s exposure to the chained schedule resulted in him engaging in challenging behavior on average at 1.00 rpm, representing a 61.58% decrease in target behaviors from initial baseline levels, and an increase in compliance with demands from baseline levels to 58.96%.

Within the chained schedule, Emily engaged in challenging behavior on average at 0.50 rpm, showing an 80.67% reduction in target challenging behaviors relative to baseline. She demonstrated an increase in compliance with demands from baseline, complying with 100.00% of demands in the chained schedule.

During the chained schedule, Liam engaged in challenging behavior on average at 0.77 rpm, representing a 77.23% decrease in target challenging behavior relative to baseline. Additionally, he demonstrated an increase in compliance with demands from baseline levels, complying with 70.53% of demands within the chained schedule.

Overall, increases in compliance and decreases in target challenging behavior maintained through demand fading sessions across all participants. Allan’s challenging behavior averaged 0.83 rpm (range: 0.00–4.36). In his final two sessions of FR5, he engaged in no target responses, reflecting a 100% decrease in challenging behavior from baseline. Additionally, he complied with an average of 94.34% demands across his final two sessions.

Emily’s challenging behavior averaged 0.39 rpm (range: 0.00–1.78) within demand fading sessions. In her final two FR5 sessions, she engaged in no target responses, also reflecting a 100% decrease in challenging behavior from baseline. She complied with an average of 88.08% of demands across her final two sessions.

Liam’s challenging behavior averaged 1.08 rpm (range: 0.00–8.10) within demand fading sessions. In his final two FR5 sessions, he engaged in 0.10 target responses per minute, representing a 96.40% decrease in challenging behavior from baseline levels. Liam complied with all demands in his final two sessions.

## 5. Discussion

The present study sought to replicate and extend the findings of Lalli et al. (1995) by systematically evaluating the effects of DRC and FCT on escape-maintained challenging behavior. Specifically, this study examined the efficacy of these interventions in isolation and in combination within a chained schedule of reinforcement. The findings contribute to the existing literature by addressing known limitations of DRC and FCT while incorporating strategies that have been demonstrated to improve treatment sustainability and generalization (e.g., use of a chained schedule, demand fading, incorporating preference; Kranak & Brown, 2023; Pelton et al., 2024).

Results for the independent implementation of DRC and FCT varied across participants, with FCT effectively reducing escape-maintained challenging behavior from baseline levels across all participants, and DRC reducing escape-maintained challenging behavior from baseline levels for two participants. Consistent with previous research (e.g., Piazza et al., 1996), the implementation of DRC increased compliance but did not reliably promote functional communication or decreased rates of challenging behavior across participants. In contrast, FCT facilitated the use of appropriate communication responses and decreased challenging behavior but failed to enhance compliance. By integrating these interventions within a chained schedule arrangement, the individual limitations of these interventions were successfully mitigated. Specifically, reinforcing compliance with access to the preferred treatment (FCT) promoted both task engagement and appropriate communication in lieu of challenging behavior, aligning with the work of Lalli et al. (1995) and extending their findings by directly assessing the independent contributions of DRC and FCT prior to their integration.

Furthermore, previous research suggests that chained schedule approaches as used in Lalli et al. (1995) may be particularly beneficial for individuals whose challenging behavior is maintained by negative reinforcement contingencies (i.e., escape or avoidance; Kranak & Brown, 2023). As noted by Kranak and Brown (2023), such approaches promote task completion within a DRC while ensuring access to breaks through an appropriate FCR. This is further supported by Greer et al. (2016), who found that embedding FCT within a structured contingency such as a chained schedule enhanced the persistence of alternative responses while reducing reliance on dense schedules of reinforcement.

This study extends prior research in several meaningful ways. First, it further documents the limitations of DRC and FCT when used in isolation, highlighting the need for combined intervention approaches. Second, it directly assessed treatment preference, revealing a clear preference for FCT over DRC for all participants. This finding has important implications for treatment adherence and long-term effectiveness, as interventions that align with individual preferences are more likely to be maintained in natural settings (Hanley, 2010). Additionally, assessing and incorporating client preference for treatment type aligns with both current best practice standards of evidence-based practice for the American Psychological Association (American Psychological Association, 2021) and ethical standards regarding responsibility in practice for behavior analysts (Behavior Analyst Certification Board, 2020). Third, by incorporating DRC and FCT into a chained schedule, this study ensured that compliance was a necessary prerequisite for reinforcement, further enhancing treatment efficacy. Finally, this study demonstrated that reinforcing compliance with access to a preferred intervention (i.e., FCT) within the chained schedule arrangement facilitated both compliance and functional communication.

The use of a chained schedule consists of a sequence of behaviors, where each component must be completed in order, and the completion of one component leads to access to the next (Ferster & Skinner, 1957). In this arrangement, the second component (access to the preferred intervention) may come to serve as a conditioned reinforcer, strengthening the behavior required to complete the first component (i.e., compliance with the initial task demand). The use of the preferred intervention as the second component capitalized on the principle of conditioned reinforcement, where stimuli that were initially neutral but had been paired with reinforcers acquire reinforcing properties (Skinner, 1953). This design provides a practical strategy for addressing escape-maintained behavior, as it links the aversive task (the first component) to access to a more preferred activity (the second component). Moreover, this arrangement allows for the gradual reduction in dense reinforcement schedules, promoting sustained behavior change while minimizing the need for continued dense reinforcement schedules.

Although the findings of this study provide valuable insights, several directions for future research remain. One important consideration is the potential for resurgence of challenging behavior during demand fading. As reinforcement schedules are systematically thinned, challenging behavior may re-emerge if alternative responses are not sufficiently reinforced (Wacker et al., 2013). Intermittent increases in challenging behavior were observed for all participants during schedule thinning sessions (e.g., Allan at sessions 68, 70, and 75; Emily at sessions 29, 34, 37, and 40; Liam at sessions 56 and 58), potentially as a result of the reduced density of reinforcement (Briggs et al., 2018; Falligant et al., 2022; Muething et al., 2021; Muething et al., 2024). Future studies should evaluate strategies to mitigate resurgence in this arrangement (Fuhrman et al., 2016).

Additionally, it should be noted that the intention of programming the terminal schedule as an FR 5 in this study was to demonstrate that schedule thinning was feasible following treatment chaining. Following completion of the study proper, terminal goals were selected based on the goals and needs of the client and their caregivers. Thus, schedule thinning continued beyond the FR 5 schedule for all participants, but occurred outside the context of the study. It should also be noted that within the schedule thinning literature, at times experimenters will include a terminal probe prior to initiating schedule thinning (e.g., going from FR 1 directly to FR 5), potentially increasing the efficiency of the schedule thinning process or eliminating the need to thin the schedule altogether (Kranak & Falligant, 2022). For the purpose of this study, we did not include a terminal probe, but future researchers should consider its inclusion.

A potential limitation of this study is that we combined multiple topographies of challenging behavior into a single response category for Allan and Liam. Although this approach may obscure potential functional differences across topographies, it is a common and accepted practice in behavior analytic research on the reduction in challenging behavior, particularly when topographies co-occur and share similar reinforcers (e.g., Helvey et al., 2023). Moreover, both participants’ target behavior came under control of the programmed contingencies, suggesting that the aggregated responses functioned as a unified response class within the context of our analysis. Nonetheless, future research may benefit from evaluating individual topographies separately to assess potential functional distinctions more precisely.

Another limitation of this study is that two of our three participants had previous experience with one of the interventions used (FCT). Although we equated the exposure sessions in which we paired the color cards with each respective intervention, this history may limit the interpretation of the preference assessment for participants Emily and Liam, given that FCT had a longer history of reinforcement. Future researchers should replicate this process while evaluating interventions that are entirely novel to the participants.

Future research should also examine the effects of different approaches to DRC, as this may impact both the success of DRC alone and the client’s preference for it. One key consideration is the type of demands used when implementing DRC. Research suggests that high-probability instructional sequences can enhance compliance within DRC interventions (Lipschultz et al., 2018). However, suppose omission criteria require extended periods of compliance before reinforcement is delivered, the effectiveness of the intervention (i.e., high p sequences) may be reduced, particularly if the delay weakens the association between compliance and reinforcement (Lattal, 2010). A second consideration is the schedule and magnitude of reinforcement in DRC. Fulton et al. (2020) compared the effectiveness of accumulated reinforcement (i.e., reinforcement delivered after multiple instances of compliance resulting in longer but less frequent breaks) and distributed reinforcement schedules (i.e., breaks provided in shorter, immediate increments following each compliant response) within DRC and found that accumulated reinforcement resulted in lower levels of challenging behavior and higher levels of compliance. Future research should assess how different approaches to DRC, including the type of demand and reinforcement schedule and magnitude of the reinforcer, may influence performance during initial intervention comparisons, as well as their impact on treatment preference and the long-term effectiveness of DRC when used alongside other intervention components.

It is important to note that although the chaining of DRC and FCT is a step toward a more assent-based model, it is not a purely assent-based approach, as there is still a requirement for compliance prior to negative reinforcement. This requirement means, in some scenarios, the individual cannot immediately opt out of non-preferred activities, even when communicating that the activity is not preferred. As a potential future direction, arranging FCT and DRC contingencies concurrently—rather than as a chain—may offer a more assent-aligned alternative. In such arrangements, task completion may be reframed not as compliance, but as voluntary participation or engagement, with the goal of minimizing the aversive aspects of the task and arranging reinforcement of sufficient quality and magnitude to make participation the preferred choice. Importantly, this approach allows participants the opportunity to opt out at any time, supporting a model of treatment that promotes autonomy, assent, and meaningful choice.

When addressing escape-maintained behavior, it is essential to incorporate FCT into compliance training to promote self-advocacy. Recently, there has been a growing emphasis within applied behavior analysis on reducing the focus on compliance training and instead prioritizing assent-based practices, ensuring that individuals are given meaningful opportunities to consent or dissent to participation in both clinical and research contexts (Morris et al., 2021). Although increasing compliance is often a primary treatment goal—and a significant, well-documented criticism of applied behavior analysis as a whole—an overemphasis on compliance alone risks reinforcing blind adherence to instructions without fostering the ability to communicate individual needs (Sandoval-Norton et al., 2019). Individuals should be taught to request breaks, modifications, or assistance as functionally appropriate alternatives to challenging behavior, thereby balancing compliance with autonomy. The chaining of DRC with FCT may help to promote self-advocacy and mitigate the generalization of blind compliance to unsafe situations.

## Data Availability

The data presented in this study are available on request from the corresponding author.
