# Correction: Ferris et al. (2025) Chaining Differential Reinforcement of Compliance and Functional Communication Training to Treat Challenging Behavior Maintained by Negative Reinforcement. *Behavioral Sciences*, *15*(7), 891

**DOI:** 10.3390/bs16030475

**Published:** 2026-03-23

**Authors:** Emily L. Ferris, Alexandra R. Howard, Eleni Baker, Daniel J. Hodge, Andrew R. Craig, Henry S. Roane, William E. Sullivan

**Affiliations:** Golisano Center for Special Needs, SUNY Upstate Medical University, Syracuse, NY 13210, USA

The authors would like to add a new author in the original publication (Ferris et al., 2025) for the following two reasons:

1. Daniel J. Hodge participated with Dr. Sullivan’s research group as a Summer Undergraduate Research Fellow in the summer of 2024, during which time he:
  - sorted through archival data to organize all relevant participant information;
  - audited data entry from raw data files;
  - developed preliminary graphs of which final graphs for the publication were based;
  - drafted a rough outline of the Introduction and Discussion that informed the final document.
2. Because of the latency between Daniel J. Hodge’s work on the manuscript and its publication, his contributions were inadvertently overlooked when the paper was submitted. Daniel J. Hodge does not have any conflicts of interest to declare, and other authors have agreed to this change in reporting.

The Correct Author Contributions Statement should read:

**Author Contributions:** Conceptualization, H.S.R. and W.E.S.; Methodology, W.E.S.; Validation, E.L.F., H.S.R. and W.E.S.; Formal analysis, E.L.F., A.R.H. and W.E.S.; Investigation, W.E.S.; Resources, H.S.R.; Data curation, E.L.F., D.J.H. and W.E.S.; Writing—original draft preparation, E.L.F., D.J.H. and A.R.H.; Writing—review & editing, E.L.F., E.B., D.J.H., A.R.C. and W.E.S.; Visualization, E.L.F.; Supervision, E.L.F., A.R.C., H.S.R. and W.E.S.; Project administration, H.S.R. and W.E.S. All authors have read and agreed to the published version of the manuscript.

The authors state that the scientific conclusions are unaffected. This correction was approved by the Academic Editor. The original publication has also been updated.

## References

[B1-behavsci-16-00475] Ferris E. L., Howard A. R., Baker E., Hodge D. J., Craig A. R., Roane H. S., Sullivan W. E. (2025). Chaining differential reinforcement of compliance and functional communication training to treat challenging behavior maintained by negative reinforcement. Behavioral Sciences.

